# Cash Transfers and Psychiatric Hospitalization for At-Risk Populations in Brazil

**DOI:** 10.1001/jamanetworkopen.2026.6571

**Published:** 2026-04-13

**Authors:** Camila Barreto Bonfim, Flávia Alves, Maurício L. Barreto, Vikram Patel, Daiane B. Machado

**Affiliations:** 1 Center of Data and Knowledge Integration for Health, Salvador, Bahia, Brazil; 2 Department of Global Health and Social Medicine, Harvard Medical School, Boston, Massachusetts

## Abstract

**Importance:**

Psychiatric disorders are a major global public health concern, highlighting the urgent need for policies to mitigate their impact, especially for at-risk populations.

**Objective:**

To investigate the association of the Brazilian conditional cash transfer program (Bolsa Familia Program [BFP]) with psychiatric disorder hospitalizations and availability of community mental health service.

**Design, Setting, and Participants:**

This cohort study was conducted during an 8-year period using linked social and health data from the 100 Million Brazilian Cohort. The participants were all individuals who were registered at CadÚnico between January 1, 2008, and December 31, 2015. Statistical analyses were conducted between August 12, 2025, and January 31, 2026.

**Exposure:**

Participation in the BFP.

**Main Outcomes and Measures:**

All psychiatric disorder hospitalizations registered on the Hospital Information System according to *International Statistical Classification of Diseases and Related Health Problems, Tenth Revision (ICD-10) *as psychiatric disorders, except for substance use disorders.

**Results:**

A total of 38 572 143 individuals (mean [SD] age, 21.66 [22.06] years; 20 661 009 [54%] female and 17 911 134 [44%] male; 188 033 [0.49%] Asian, 2 238 344 [5.80%] Black, 280 859 [0.73%] Indigenous, 22 123 435 [57.36%] Pardo [includes individuals with Black or mixed ancestry, including European, African, and Indigenous heritage], and 12 749 758 [33.05%] White) were included in the analysis. A total of 56 646 individuals (0.1%) had a psychiatric disorder hospitalization during the study period. Individuals who received a cash transfer from the BFP had a 23% lower risk of psychiatric disorders hospitalization (hazard ratio [HR], 0.77; 95% CI, 0.76-0.79). The association was stronger among Pardo (HR, 0.72; 95% CI, 0.69-0.74) and Black (HR, 0.78; 95% CI, 0.72-0.85) individuals who lived in the poorest municipalities (HR, 0.67; 95% CI, 0.63-0.71) regardless community mental health care.

**Conclusions and Relevance:**

In this cohort study of the 100 Million Brazilian Cohort, a national cash transfer program was associated with lower risk of psychiatric hospitalizations, especially among at-risk populations who experience health inequalities.

## Key Points

**Question:**

Is a large Brazilian conditional cash transfer program associated with lower risk of psychiatric hospitalizations, especially among at-risk populations?

**Findings:**

In this cohort study using administrative databases linked to the baseline of the 100 Million Brazilian Cohort, a large Brazilian conditional cash transfer program was associated with 23% lower risk of psychiatric hospitalizations. This association was stronger among Pardo and Black individuals, who have the lowest income among individuals living in Brazilian municipalities, regardless of psychosocial care center availability.

**Meaning:**

This study’s results suggest that poverty alleviation programs may reduce psychiatric hospitalizations in high-risk populations.

## Teaser

This cohort study examines whether a conditional cash transfer program is associated with fewer psychiatric disorder hospitalizations in an at-risk Brazilian population.

## Introduction

Psychiatric disorders represent a major component of the global burden of disease.^1^ They are among the primary causes of disability worldwide, accounting for 10 to 20 years lived with disabilities.^2^ More than 1 billion people worldwide are estimated to experience psychiatric disorders, representing a major public health problem.^2^ At the household level, approximately 7 of every 1000 families include at least 1 individual living with a psychiatric disorder.^1^

Although psychiatric disorders contribute substantially to the global burden of disease, access to mental health services remains limited, particularly in low- and middle-income countries.^2^ Only approximately 30% of individuals with severe mental disorders receive treatment.^2^ Limited access to timely care may lead to worsening conditions and, consequently, to psychiatric hospitalization—an undesirable outcome that often reflects gaps in community-based care and adverse social determinants.^2,3^

Psychiatric disorders are associated with a range of risk factors; however, poverty has emerged as a prominent focus of research in recent years.^4^ Living in conditions of poverty has been associated with increased exposure to multiple stressors, thereby heightening the risk of severe psychiatric disorders.^4,5^ In this context, poverty alleviation strategies have been explored as potential interventions to address this substantial public health concern.^6,7,8^

Cash transfer programs are primarily focused on alleviating poverty and extreme poverty, and evidence suggests that they confer substantial mental health benefits.^6,9,10,11^ Studies have explored the role of cash transfer programs in mental health outcomes, especially for common mental disorders,^7,12,13^ such as depressive symptoms^9,10,14^ and anxiety.^14,15^ By reducing poverty, cash transfer programs have the potential to mitigate financial stress, enhance the sense of self-efficacy and self-control, promote access to health care and education, and facilitate social inclusion and citizenship.^5^

Despite the increasing body of evidence on the effects of cash transfer programs on mental health, findings remain inconsistent.^5^ Although some studies have demonstrated a protective effect of cash transfer programs on mental health outcomes,^9,10,11,14^ others have reported heterogeneous findings, including partial benefits in specific subgroups,^13,15,16,17,18,19,20^ null effects,^21^ or even potential adverse impacts.^22,23,24^ These studies, in general, have small samples, including subgroups such as children or adults only, focus on common mental disorders, and rely on primary data collection, which may be biased by social desirability.

There remains a need for studies examining the association between the BFP and severe psychiatric disorders among socially at-risk populations exposed to health inequities, particularly in low- and middle-income countries. Residents of poorer municipalities generally face restricted access to health care services, which heightens their risk of morbidity and mortality.^25^ In Brazil, these disparities are particularly pronounced among Black and multiracial populations, who experience reduced access to health care, lower income levels, and disproportionate exposure to the mental health consequences of structural racism.^26,27^

Therefore, more robust studies that examine the impact of these interventions on mental disorders, particularly severe conditions that need hospitalization using nationally representative data, are clearly needed. Furthermore, it remains necessary to investigate further the effects of cash transfer programs on severe mental health, particularly among at-risk populations, considering the impact of poverty reduction on these outcomes. This study aims to investigate the association of the BFP with psychiatric disorder hospitalizations overall and stratified by race, municipal level of material deprivation, and availability of community mental health services in Brazilian municipalities. We hypothesize that the BFP is associated with lower risk of psychiatric disorder hospitalizations and that this association is more pronounced in at-risk populations as well as among individuals with limited access to community mental health services.

## Methods

### Study Design, Data Sources, and Dataset Linkage

This longitudinal cohort study used different administrative databases linked to the baseline of the 100 Million Brazilian Cohort, a population-based dynamic cohort including individuals who applied for social benefits and use the public health system in Brazil.^28^ This baseline draws on records from more than 131 million individuals registered between 2001 and 2018 in CadÚnico, Brazil’s central registry for all individuals and families applying for any social protection programs.^29^ It contains a large amount of socioeconomic and demographic data on the applicant and all family members. Eligibility for access to be registered at CadÚnico requires a per capita income of up to half the minimum wage or a total household income of up to 3 minimum wages.^28^

We linked the baseline with the Hospitalisation Information System (SIH) and the Bolsa Família Program (BFP) payroll database.^30^ The SIH is Brazil’s national database of hospital admissions funded by the public health care system, which covers approximately 75% of the population and includes both general and specialized hospitals.^31,32^ Hospital data are recorded by health care professionals using standardized forms and the *International Statistical Classification of Diseases and Related Health Problems, Tenth Revision (ICD-10) *codes to identify primary and secondary diagnoses.^33^ The BFP payroll database is the system that records information on the monthly benefits paid by the program for each family.^30^

All data were linked using the CIDACS-RL, an open-source record linkage algorithm developed by the Center for Data and Knowledge Integration for Health (CIDACS).^34^ BFP data were deterministically linked to the 100 Million Brazilian Cohort using the Social Identification Number. Additionally, a linkage algorithm was applied to match records between the SIH and CadÚnico databases based on 5 common variables: beneficiary’s name, mother’s name, sex, municipality code of residence, and date of birth (eMethods 1 in Supplement 1). Additional details about the linkage process have been previously published^34^ (eFigure 1 in Supplement 1). This study was approved by the ethics committees of the Gonçalo Muniz Institute at the Oswaldo Cruz Foundation. Data were housed on secure servers within the Big Data Integrated Platform of the CIDACS. Because the analytical dataset contained no personally identifiable information, the requirement for informed consent was waived by the ethics committees. This study followed the Strengthening the Reporting of Observational Studies in Epidemiology (STROBE) reporting guideline for cohort studies.

### Participants

The study population consists of 38 649 094 individuals who were registered at CadÚnico from January 1, 2008, to December 31, 2015, when the hospitalization data were available. We excluded 884 individuals who had repeated registration, 29 197 who died before CadÚnico registration, 43 429 who were hospitalized before entering the cohort, 3408 who received BFP after hospitalization, and 33 who had the same entry and exit date in the cohort (Figure).

**Figure.**
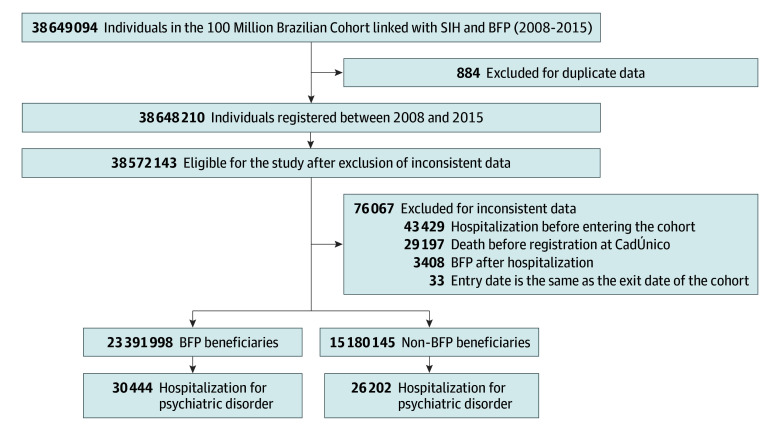
Flowchart of the Study Population BFP indicates Bolsa Família Program; SIH, Hospitalisation Information System.

### Exposure, Outcome, and Follow-Up

#### Exposure

BFP is one of the largest social programs implemented by a government worldwide.^35^ It was designed to reduce poverty and extreme poverty in a continental-sized, middle-income country with millions living below the poverty line.^36^ However, its impacts have gone beyond its initial goals, contributing to a range of positive trends in different health outcomes, including lower suicide rates.^8^ To be eligible for BFP, a family must be registered in CadÚnico and be classified as living in poverty (monthly per capita household income of less than R $154 [US $39] in 2015) or extreme poverty (monthly per capita household income of less than R $77 or US $19 in 2015).^28^

Additional variable benefits of R $35 to $42 (US $9-$11) may be granted for each child, adolescent, pregnant woman, or breastfeeding mother in the household.^28^ The program also requires children younger than 7 years to follow the vaccination schedule and receive nutritional monitoring, pregnant women to attend prenatal care, and school attendance for children, adolescents, and youth.^36^ We considered individuals who received the benefit during the period as beneficiaries and those who did not as nonbeneficiaries.

#### Outcome

Our outcome was psychiatric disorder hospitalizations registered on the SIH. We considered any psychiatric disorder hospitalization and all admissions registered on the SIH with a primary or secondary diagnosis of psychiatric disorders (*ICD-10* codes F00-F99).^33^ We excluded hospitalizations for substance use disorders (*ICD-10* codes F10-19), considering evidence previously explored in this cohort.^37^

#### Follow-Up

For the BFP beneficiary subset, these individuals were followed up from the time they started to receive the BFP benefit to the time their follow-up ended due to death by any cause, the first hospitalization for psychiatric disorder after the follow-up started, time they stopped receiving BFP benefits, or December 31, 2015, when the follow-up period finished. For the nonbeneficiary subset (ie, individuals who did not receive BFP benefit during our follow-up time), the follow-up started when these individuals were registered on CadÚnico and ended due to death by any cause, the first hospitalization for psychiatric disorder, time they became a BFP beneficiary, or the end of the follow-up period on December 31, 2015.

### Statistical Analysis

First, we performed a descriptive analysis comparing the groups of beneficiaries and nonbeneficiaries of BFP using the propensity score (PS) method, with 2-sided *P* < .05 considered statistically signficant (eMethods 2 in Supplement 1). For this purpose, we estimated the PS by logistic regression with the following baseline covariates already associated with the BFP according to literature review: sex, age, educational level, self-reported race (Asian, Black, Indigenous, Pardo, or White), location of residence, household characteristics (water supply, waste, sanitation, and construction materials), isolation, Brazilian regions of residence, and year of CadÚnico registration (eTable 1 in Supplement 1).^8,38,39,40^ Racial categories followed the terms of the Brazilian Institute of Geography and Statistics, reflecting national classifications of race.^41^ The term Pardo (Portuguese for “brown” or “mixed”) denotes individuals with Black or mixed ancestry, including European, African, and Indigenous heritage.^42^ Self-reported race (Asian, Black, Indigenous, Pardo, or White) data were collected to assess potential racial inequities in psychiatric hospitalization. In Brazil, poverty and structural inequalities disproportionately affect Black and Pardo populations, who also constitute a large share of beneficiaries of the BFP.^26^ Therefore, race was included to test the hypothesis that these socially disadvantaged populations would have a lower likelihood of psychiatric hospitalization. We then assessed the common support graph (eFigure 2 in Supplement 1) and compared the range of PSs among BFP and non-BFP groups (eTables 2 and 3 in Supplement 1).^43^

Second, we estimated PS weights using the inverse probability of treatment weighting (IPTW) approach to balance differences among covariates in the BFP and non-BFP groups.^43^^,^ Beneficiaries were given weights equal to the inverse of their PSs (1/PS), whereas those who were not beneficiaries were given weights equal to the inverse of 1 minus their PSs (PS/[1 – PS]). To enhance estimation accuracy and precision, IPTW was applied along with weight truncation at the 99th percentile.^40,43,44^

Third, covariate imbalance between groups was identified based on absolute differences in category proportions exceeding 0.1 both before and after IPTW weighting, reflecting the categorical nature of our covariates.^43^ We also estimated cumulative distribution balancing plots by covariates before and after IPTW^39,45^ (eFigure 3 in Supplement 1).

Fourth, we calculated the incidence rate for hospitalization for psychiatric disorders for both groups starting from the date of hospitalization and ending in 2015. Person-year per 100 000 individuals was used as a denominator.

Fifth, Cox proportional hazards regression was estimated using hazard ratios (HRs) adjusted by IPTW^46^ and 95% CIs to assess the association between BFP and lower hospitalization rates. To test our hypothesis that the association between BFP and psychiatric hospitalizations is stronger among at-risk populations, we conducted stratified multivariate Cox proportional hazards regression models with extended specification,^47^ including stratifications by race or skin color and the Brazilian Deprivation Index (IBP).^48^

Sixth, given the established role of psychosocial care services in Brazil,^49^ we examined whether psychosocial care center availability modified the association between BFP participation and psychiatric hospitalizations. Material deprivation level was assessed using population-weighted tertiles of the 2010 IBP,^48^ which is calculated based on the proportion of households with a per capita income below half the minimum wage, the proportion of individuals aged 7 years who have illiteracy, and the proportion of residents living in inadequate housing. The availability of psychosocial care centers was defined by their documented presence within the municipality during the study period, following previously published criteria.^3^ All psychosocial care center modalities were considered in the analysis. Lastly, we assessed the robustness of our findings using multiple approaches (eMethods 3 in Supplement 1). All analyses were conducted from August 12, 2025, to January 31, 2026, using Stata software, version 15.0 (StataCorp).

## Results

Of the 38 572 143 individuals (mean [SD] age, 21.66 [22.06] years; 20 661 009 [54%] female and 17 911 134 [44%] male; 188 033 [0.49%] Asian, 2 238 344 [5.80%] Black, 280 859 [0.73%] Indigenous, 22 123 435 [57.36%] Pardo, and 12 749 758 [33.05%] White) in the study, 56 646 (0.1%) had a first psychiatric disorder hospitalization during the follow-up period. A total of 23 391 998 (60.6%) were BFP beneficiaries, of whom 30 444 (53.7%) were hospitalized with psychiatric disorders. Among the 15 180 145 nonbeneficiaries (39.4%), 26 202 (46.3%) were hospitalized with psychiatric disorders. At the cohort baseline, differences were observed between the 2 groups in sociodemographic and socioeconomic characteristics; however, after applying IPTW, these differences became balanced, indicating adequate comparability between the groups of BFP beneficiaries and nonbeneficiaries (Table 1). The incidence rate of psychiatric hospitalization was lower among beneficiaries (27.09 [95% CI, 26.78-27.41] per 100 000 population) compared with nonbeneficiaries (34.54 [95% CI, 33.88-35.23] per 100 000 population).

**Table 1.** Characteristics of the BFP Nonbeneficiaries and Beneficiaries Before and After IPTW, 2008 to 2015

Characteristic	Before IPTW, No. (%) (n = 38 572 143)			After IPTW, No. (%) (n = 34 864 735)		
	BFP (n = 23 391 998)	Non-BFP (n = 15 180 145)		BFP (n = 21 230 441)	Non-BFP (n = 13 634 294)	
Sex						
Male	10 660 899 (45.6)	7 250 235 (47.8)	0.04	9 693 812 (45.4)	6 471 566 (47.5)	−0.01
Female	12 731 099 (54.4)	7 929 910 (52.2)		11 590 629 (54.6)	7 162 728 (52.5)	
Age group, y						
<10	11 565 788 (49.4)	4 711 901 (31.0)	−0.59	10 549 572 (49.7)	4 239 320 (31.1)	0.02
10-24	4 698 430 (20.1)	2 176 063 (14.3)		4 303 534 (20.3)	195 039 (14.5)	
25-59	6 725 205 (28.8)	5 595 879 (36.9)		6 014 555 (28.3)	4 954 002 (36.3)	
>60	402 575 (1.7)	2 696 302 (17.8)		362 780 (1.7)	2 465 933 (18.1)	
Educational level (years of education)						
Never studied	9 760 907 (41.7)	4 856 864 (32.0)	−0.29	8 749 635 (41.2)	4 298 412 (31.5)	0.03
Preschool	1 626 347 (7.0)	696 253 (4.6)		1 535 887 (7.2)	653 440 (4.8)	
Primary school or less (≤5 y)	5 106 795 (21.8)	3 812 831 (25.1)		4 703 637 (22.2)	3 492 402 (25.6)	
Junior high school (6- 10 y)	3 766 058 (16.1)	2 052 118 (13.5)		3 500 127 (16.5)	1 865 559 (13.7)	
High school (10-12 y)	2 986 005 (12.8)	3 294 248 (21.7)		2 642 920 (12.4)	2 926 521 (21.4)	
College or university (≥13 y)	109 671 (0.5)	443 067 (2.9)		98 235 (0.5)	397 960 (2.9)	
Missing data	36 215 (0.2)	24 764 (0.2)		NA	NA	
Race						
Asian	101 191 (0.4)	86 842 (0.6)	0.23	88 010 (0.4)	79 704 (0.6)	0.02
Black	1 407 979 (6.0)	830 365 (5.5)		1 306 160 (6.2)	773 900 (5.7)	
Indigenous	224 260 (1.0)	56 599 (0.4)		156 617 (0.7)	33 600 (51.6)	
Pardo^^a^^	14 405 544 (61.6)	7 717 891 (50.8)		13 188 708 (62.1)	7 038 840 (51.6)	
White	6 810 468 (29.1)	5 939 290 (39.1)		6 490 946 (30.6)	5 708 250 (41.9)	
Missing data	442 556 (1.9)	545 158 (3.6)		NA	NA	
Location of residence						
Rural	4 746 917 (20.3)	2 186 787 (14.4)	0.17	3 866 661 (18.2)	1 673 582 (12.3)	0.04
Urban	18 613 150 (79.6)	12 988 421 (85.6)		17 363 780 (81.8)	11 960 712 (87.7)	
Missing data	31 931 (0.1)	4937 (<0.1)		NA	NA	
Brazilian regions						
Southeast	8 466 468 (36.2)	5 970 433 (39.3)	0.01	7 975 645 (37.6)	5 557 781 (40.8)	0.03
Northeast	7 839 226 (33.5)	3 906 616 (25.7)		6 900 020 (32.5)	3 350 297 (24.6)	
Central-West	1 776 041 (7.6)	1 585 874 (10.4)		1 644 728 (7.8)	1 430 333 (10.5)	
South	2 045 045 (8.7)	2 310 921 (15.2)		1 893 290 (8.9)	2 127 936 (15.6)	
North	3 265 218 (14.0)	1 406 301 (9.3)		2 816 758 (13.3)	1 167 947 (8.6)	
Household characteristics						
Water supply						
Public network (running water)	16 545 090 (70.7)	12 123 291 (79.9)	0.24	15 945 936 (75.1)	11 544 453 (84.7)	0.06
Well, natural sources, or other	6 069 908 (26.0)	2 490 049 (16.4)		5 284 505 (24.9)	2 089 841 (15.3)	
Missing data	777 000 (3.3)	566 805 (3.7)		NA	NA	
Waste						
Public collection system	18 090 879 (77.3)	12 940 021 (85.2)	0.24	17 424 613 (82.1)	12 122 203 (88.9)	0.06
Burned, buried, outdoor disposal, or other	4 524 127 (19.3)	1 673 319 (11.0)		3 805 828 (17.9)	1 512 091 (11.1)	
Missing data	776 992 (3.3)	566 805 (3.7)		NA	NA	
Sanitation public network	11 169 291 (47.8)	8 398 967 (55.3)		10 904 961 (51.4)	8 076 469 (59.2)	
Septic tank	3 059 042 (13.1)	2 010 314 (13.2)	0.21	3 003 841 (14.2)	1 931 053 (14.2)	0.05
Homemade septic tank	5 562 476 (23.8)	3 299 753 (21.7)		5 470 791 (25.8)	3 179 368 (23.3)	
Ditch or other	1 873 077 (8.0)	459 107 (3.0)		1 850 848 (8.7)	447 404 (3.3)	
Missing data	1 728 112 (7.4)	1 012 004 (6.7)		NA	NA	
Construction materials						
Bricks or cement	18 086 326 (77.3)	12 825 966 (84.5)	0.21	17 268 855 (81.3)	12 122 203 (88.9)	0.06
Wood, other plant materials, or other	4 528 680 (19.4)	1 787 374 (11.8)		3 961 586 (18.7)	1 512 091 (11.1)	
Missing data	776 992 (3.3)	566 805 (3.7)		NA	NA	
Isolation						
Lives with someone else	22 511 438 (96.2)	13 138 366 (86.6)	−0.35	20 526 266 (96.7)	11 902 116 (87.3)	−0.01
Lives alone	880 560 (3.8)	2 041 779 (13.4)		704 175 (3.3)	1 732 178 (12.7)	
Year of registration on CadÚnico						
2008	3 944 749 (16.9)	742 264 (4.9)	−0.87	3 809 195 (17.9)	710 121 (5.2)	−0.04
2009	3 916 878 (16.7)	812 326 (5.4)		3 781 444 (17.8)	775 615 (5.69)	
2010	4 110 268 (17.6)	1 160 363 (7.6)		3 951 341 (18.6)	1 113 686 (8.2)	
2011	2 589 141 (11.1)	1 466 487 (9.7)		2 327 310 (11.0)	1 380 889 (10.1)	
2012	3 578 423 (15.3)	2 980 852 (19.6)		3 066 873 (14.4)	2 747 625 (20.2)	
2013	2 423 609 (10.4)	2 533 624 (16.7)		1 813 752 (8.5)	1 843 372 (13.5)	
2014	1 866 796 (8.0)	3 002 787 (19.8)		1 619 310 (7.6)	2 756 146 (20.2)	
2015	962 134 (4.1)	2 481 442 (16.4)		861 216 (4.1)	2 306 840 (16.9)	

Abbreviations: BFP, Bolsa Família Program; IPTW, inverse probability of treatment weighting; NA, not applicable.

^a^ Pardo includes individuals with Black or mixed ancestry, including European, African, and Indigenous heritage.

When estimated with IPTW, considering sex, age, race, educational level, household characteristics (water supply, waste, sanitation, and construction materials), living alone, Brazilian region, location of residence, and year of CadÚnico registration using the Cox proportional hazards regression model, receipt of BFP cash transfer was associated with a 23% lower risk of psychiatric disorder hospitalization (HR, 0.77; 95% CI, 0.76-0.79). The subgroup analysis found this association was stronger among Pardo (HR, 0.72; 95% CI, 0.69-0.74) and Black (HR, 0.78; 95% CI, 0.72-0.85) groups (Table 2).

**Table 2.** Association of Bolsa Familia Program Participation With Psychiatric Hospitalizations by Subgroups, 2008 to 2015

Subgroup	HR (95% CI)^a^	*P* value
Race		
Asian	0.83 (0.62-1.11)	.22
Black	0.78 (0.72-0.85)	<.001
Indigenous	0.55 (0.30-1.01)	.056
Pardo	0.72 (0.69-0.74)	<.001
White	0.87 (0.84-0.90)	<.001
Brazilian Deprivation Index (tertile)		
Low deprivation	0.86 (0.84-0.89)	<.001
Medium deprivation	0.80 (0.77-0.83)	<.001
High deprivation	0.67 (0.63-0.71)	<.001
Availability of psychosocial care center in the municipality		
No	0.80 (0.76-0.84)	<.001
Yes	0.77 (0.75-0.79)	<.001

Abbreviation: HR, hazard ratio.

^a^ The HRs were estimated with inverse probability of treatment weighting given sex, age, race, educational level, household characteristics (water supply, waste, sanitation, and construction materials), living alone, Brazilian region, location of residence, and year of CadÚnico registration.

The association of BFP with lower risk of psychiatric disorder hospitalizations among beneficiaries was stronger with higher municipal deprivation indexes, showing a dose-response association. Psychiatric hospitalizations among beneficiaries were reduced by 14% in municipalities with lower levels of deprivation (HR, 0.86; 95% CI, 0.84-0.89), whereas in highly deprived municipalities, the reduction reached 33% (HR, 0.67; 95% CI, 0.63-0.71). When considering the availability of CAPS across municipalities, the association was observed among individuals living in municipalities with psychosocial care center availability and individuals living without psychosocial care center availability, being slightly stronger in municipalities with availability (HR, 0.77; 95% CI, 0.75-0.79) (Table 2). All of the sensitivity analyses supported these findings (eTables 4-11 and eFigure 4 in Supplement 1).

## Discussion

Using a nationwide cohort of 38 572 143 individuals with 56 646 psychiatric disorder hospitalizations, this study found that being a BFP recipient was associated with a 23% lower risk of psychiatric disorder hospitalizations. The association was stronger particularly among Pardo and Black individuals as well as among the poorest individuals living in Brazilian municipalities, regardless of psychosocial care center availability.

Our findings on the BFP, a policy focused on poverty reduction, and its association with lower hospitalization rates for mental disorders reinforce the relationship between poverty and mental health.^4,6,7,12^ This was particularly evident among the socioeconomically most at risk because the reduction in psychiatric hospitalizations was observed in the poorest and most at-risk groups, which were observed in other studies.^4,20,40^ The most pronounced association between BFP and reduced psychiatric hospitalization was observed in most deprived municipalities and among Pardo and Black individuals, a population historically marginalized in Brazil in terms of social and economic opportunities as well as access to health care.^50^ This finding is particularly important, given that structural racism produces adverse effects on the mental health of this population^26^ as well as highlights the importance of identifying racial disparities to promote equity in access to mental health care.^51^

Several mechanisms have been identified to explain how cash transfer programs have the potential to break psychiatric disorders, particularly those with conditionalities attached. Such benefits may alleviate household financial stress, thereby reducing the likelihood of family conflicts and improving overall well-being.^6,12,52,53^ Conditionalities, in turn, can encourage individuals to seek greater health care and increase school attendance among children, both of which are associated with improvements in mental health.^5^ The biopsychosocial model is a comprehensive framework for explaining the association between cash transfer programs and mental health.^54^ This model encompasses multiple interactions between biological factors and life-course experiences, within which cash transfers may play a role in modifying the pathway between poverty and mental health.^53^

The effects of these programs can be observed at multiple levels.^5^ At the individual level, they may enhance self-efficacy, hope, internal locus of control, and self-esteem through strengthening social capital. Children may also be encouraged to attend school and take care of their health, leading to improved academic skills and physical health.^5^ At the family level, by improving financial conditions, parents may increase both the quantity and quality of interactions with their children because reduced financial stress and improved mental health allow for more time and a more supportive family environment. Consequently, parents are better positioned to encourage their children to attend school and maintain their health.^5^ At the community level, the effects may be perceived more indirectly through improvements in mental health resulting from increased use of mental health services as well as enhanced community-level interactions facilitated by schools and health services.^5^ Overall, although these programs are primarily designed to alleviate poverty, they have demonstrated significant effects on mental health, particularly among individuals with greater risk who lived health inequalities.^4,20,54^

The findings regarding the association between the BFP and psychosocial care center availability suggest that a poverty alleviation program may have had a greater impact on reducing psychiatric hospitalizations than the mere presence of community-based mental health services. Nonetheless, the importance of integrating public policies to address severe mental disorders should be stimulated.^4,17,40^ The Brazilian Psychosocial Care Network comprises different levels of care, including psychosocial care centers, hospitals, and primary health care units.^49^ Since the psychiatric reform, psychosocial care centers have served as the central community-based setting for severe mental disorders, whereas hospitals address acute cases, with longitudinal follow-up conducted at psychosocial care centers and primary care facilities.^3,49^ Our findings suggest a potential association across these care levels because BFP conditionalities require families with children and women to attend regular follow-ups in primary health care units.^28^

### Strengths and Limitations

Our study’s strengths include nationwide linked datasets with a large sample size of more than 36 million people, thus allowing the application of robust methods that would be unfeasible within the scope of primary studies. The robust sample size also enables subgroup analyses of historically vulnerable populations, underscoring the need for increased investment in mental health policies for these subgroups. In addition, relying on nationwide hospitalization records as the primary outcome provides a more objective measure than the self-reported psychiatric disorder adopted in previous studies,^9,10,18^ thereby reducing biases related to underreporting, recall, and social desirability.

However, our study has some limitations. This study reflects only acute or severe mental disorder cases because it is based on psychiatric hospitalizations. Many mental health cases are managed in psychosocial care centers, the Brazilian community mental health services, potentially reducing hospital use. Findings may also be affected by unequal geographic distribution of health care facilities, particularly hospitals. Although we accounted for key sociodemographic confounders, residual confounding may persist because the linked routine datasets did not capture important factors related to treatment adherence, such as behavioral characteristics (eg, alcohol or other substance use) or aspects of health service access and quality (eg, distance to facilities or practitioner competence). Another aspect to be considered concerns the heterogeneous effects observed across subgroups. Future studies specifically designed and adequately powered to investigate effect modification in further subgroups are warranted. Additionally, the findings are generalizable only to the poorest part of the Brazilian population because the 100 Million Brazilian Cohort includes individuals enrolled in the social protection programs registry (CadÚnico). Indeed, this population exhibits sociodemographic and socioeconomic characteristics that are different from those of the other part of the Brazilian population.^28^ Finally, although 75% of the Brazilian population is estimated to access public hospitals and because this study focuses on the poorest segment of the Brazilian population, which relies almost exclusively on the public health system,^32^ the associations reported here become more valid and generalizable to this and other similar populations.

## Conclusions

In this cohort study of the 100 Million Brazilian Cohort, we found that receipt of a BFP cash transfer reduced the odds of psychiatric disorder hospitalizations, especially for more at-risk populations. Our findings suggest that a policy targeting poverty alleviation may help reduce severe mental disorder hospitalizations, particularly among the poorest populations and Black and Pardo individuals and regardless of psychosocial care center availability in their municipalities. Therefore, based on the evidence from our study, we recommend that poverty alleviation programs, such as the BFP, be considered not only as vital instruments for enhancing the well-being of low-income families in Brazil but also as integral elements of mental health care.

## Notes

Supplement 1. **eMethods 1. **Linkage procedures **eFigure 1**. ROC curve of the 100 million Brazilian Cohort and SIH (2001-2018) linkage Source: Developed by the CIDACS Data Production Center. **eMethods 2. **Propensity score: definition, estimation, summary, and support graphs **eTable 1**. Logistic regression to estimate propensity scores for receiving Bolsa Familia according to covariables, N= 34,864,735 **eFigure 2**. Distribution of the propensity score in the sample, 2008-2015 **eFigure 3**. Cumulative Distribution Balancing Plot - Balance between variables before and after IPTW, 2008-2015 **eTable 2**. Propensity score description in accordance with the confounding covariates observed, Brazil, 2008 to 2015, N= 34,864,735 **eTable 3**. Propensity score description in accordance with the confounding covariates observed after exclusion of individuals with extreme values (<0.1 - >0.9), Brazil, 2008 to 2015, N= 29,909,313 **eMethods 3. **Sensitivity analyses **eTable 4**. Incidence rate ratio of Bolsa Família Program participation with psychiatric hospitalizations, 2008-2015. **eTable 5**. ATT of psychiatric hospitalizations for BFP receipt between 2008 and 2015 using Kernel matching **eTable 6.** Association of Bolsa Família Program participation with psychiatric hospitalizations adjusted for kernel matching, 2008-2015. **eTable 7**. Crude and adjusted association of Bolsa Família Program participation with psychiatric hospitalizations, 2008-2015. **eTable 8**. Association of Bolsa Família Program participation with psychiatric hospitalizations adjusted for IPTW after exclusion of individuals with Propensity Score extreme values (<0.1 - >0.9), 2008-2015. N= 29,909,313 **eTable 9.** Association of Bolsa Família Program participation with psychiatric hospitalizations adjusted for IPTW after exclusion of individuals hospitalized within the first year of registration in the CadÚnico, 2008-2015. N= 34,707,794 **eTable 10. **Information criteria–based model comparison evaluating the inclusion of an interaction term **eTable 11. **Proportional hazards (PH) assumption test **eFigure 4. **Kaplan-Meier curve representing time to psychiatric hospitalization by BFP
eReferences

Supplement 2.
Data Sharing Statement
