# Supplementary material for: Cash Transfers and Psychiatric Hospitalization for At-Risk Populations in Brazil
Source: JAMA Netw Open. 2026 Apr 13;9(4):e266571. doi: 10.1001/jamanetworkopen.2026.6571 (PMC13077514; doi:10.1001/jamanetworkopen.2026.6571)
Supplement: Supplement 1. — eMethods 1. Linkage procedures eFigure 1. ROC curve of the 100 million Brazilian Cohort and SIH (2001-2018) linkage Source: Developed by the CIDACS Data Production Center. eMethods 2. Propensity score: definition, estimation, summary, and support graphs eTable 1. Logistic regression to estimate propensity scores for receiving Bolsa Familia according to covariables, N= 34,864,735 eFigure 2. Distribution of the propensity score in the sample, 2008-2015 eFigure 3. Cumulative Distribution Balancing Plot - Balance between variables before and after IPTW, 2008-2015 eTable 2. Propensity score description in accordance with the confounding covariates observed, Brazil, 2008 to 2015, N= 34,864,735 eTable 3. Propensity score description in accordance with the confounding covariates observed after exclusion of individuals with extreme values (<0.1 - >0.9), Brazil, 2008 to 2015, N= 29,909,313 eMethods 3. Sensitivity analyses eTable 4. Incidence rate ratio of Bolsa Família Program participation with psychiatric hospitalizations, 2008-2015. eTable 5. ATT of psychiatric hospitalizations for BFP receipt between 2008 and 2015 using Kernel matching eTable 6. Association of Bolsa Família Program participation with psychiatric hospitalizations adjusted for kernel matching, 2008-2015. eTable 7. Crude and adjusted association of Bolsa Família Program participation with psychiatric hospitalizations, 2008-2015. eTable 8. Association of Bolsa Família Program participation with psychiatric hospitalizations adjusted for IPTW after exclusion of individuals with Propensity Score extreme values (<0.1 - >0.9), 2008-2015. N= 29,909,313 eTable 9. Association of Bolsa Família Program participation with psychiatric hospitalizations adjusted for IPTW after exclusion of individuals hospitalized within the first year of registration in the CadÚnico, 2008-2015. N= 34,707,794 eTable 10. Information criteria–based model comparison evaluating the inclusion of an interaction term eTable 11. Proportional hazar [file jamanetwopen-e266571-s001.pdf]

Supplemental Online Content

Bonfim CB, Alves F, Barreto ML, Patel V, Machado DB. Cash transfers and psychiatric hospitalization for at-risk populations in Brazil. *JAMA Netw Open*. 2026;9(4):e266571. doi:10.1001/jamanetworkopen.2026.6571

eMethods 1. Linkage procedures
eFigure 1. ROC curve of the 100 million Brazilian Cohort and SIH (2001-2018) linkage Source: Developed by the CIDACS Data Production Center.
eMethods 2. Propensity score: definition, estimation, summary, and support graphs
eTable 1. Logistic regression to estimate propensity scores for receiving Bolsa Familia according to covariables, N= 34,864,735
eFigure 2. Distribution of the propensity score in the sample, 2008-2015
eFigure 3. Cumulative Distribution Balancing Plot - Balance between variables before and after IPTW, 2008-2015
eTable 2. Propensity score description in accordance with the confounding covariates observed, Brazil, 2008 to 2015, N= 34,864,735
eTable 3. Propensity score description in accordance with the confounding covariates observed after exclusion of individuals with extreme values (<0.1 - >0.9), Brazil, 2008 to 2015, N= 29,909,313
eMethods 3. Sensitivity analyses
eTable 4. Incidence rate ratio of Bolsa Família Program participation with psychiatric hospitalizations, 2008-2015.
eTable 5. ATT of psychiatric hospitalizations for BFP receipt between 2008 and 2015 using Kernel matching
eTable 6. Association of Bolsa Família Program participation with psychiatric hospitalizations adjusted for kernel matching, 2008-2015.
eTable 7. Crude and adjusted association of Bolsa Família Program participation with psychiatric hospitalizations, 2008-2015.
eTable 8. Association of Bolsa Família Program participation with psychiatric hospitalizations adjusted for IPTW after exclusion of individuals with Propensity Score extreme values (<0.1 - >0.9), 2008-2015. N= 29,909,313
eTable 9. Association of Bolsa Família Program participation with psychiatric hospitalizations adjusted for IPTW after exclusion of individuals hospitalized within the first year of registration in the CadÚnico, 2008-2015. N= 34,707,794
eTable 10. Information criteria–based model comparison evaluating the inclusion of an interaction term
eTable 11. Proportional hazards (PH) assumption test
eFigure 4. Kaplan-Meier curve representing time to psychiatric hospitalization by BFP
eReferences

This supplemental material has been provided by the authors to give readers additional information about their work.

© 2026 Bonfim CB et al. *JAMA Network Open.*

eMethods 1. Linkage procedures

The data from the 100 Million Brazilian Cohort was linked with records of the Bolsa Família Program (BFP) payments and the Hospital Information System (SIH). The linkage between the cohort and BFP utilized a deterministic approach, relying on a common correspondence key between the two databases (social identification number). Subsequently, record linkage using CIDACS-RL[1 2], a tool for linking individual records in two stages using identifiers, was employed to connect information from the 100 Million Brazilian Cohort (2001-2018) with SIH (2008-2018). This process involved utilizing variables such as the name, mother's name, date of birth, sex, and municipality of residence[1 2]. The initial stage comprised deterministic linkage of five variables, followed by the second stage based on a similarity index derived from these variables[2]. To assess the accuracy of the linkage, a manual verification of a randomly selected sample was performed and evaluated through a receiver operating characteristic curve, considering sensitivity and specificity indexes (eFigure 1). All linkage procedures were executed at the Center for Data and Knowledge Integration for Health (CIDACS)/ Fiocruz[3], within a stringent data protection environment and in adherence to ethical and legal standards[4].

Linkage between 100 million Brazilian Cohort and SIH:

- Indexed database (the largest): 100 million cohort; period 2001-2018; number of records 131,697,800.
- Search database (smaller database): SIH; period 2008-2018; number of records 27,858,929.
- Variables used: Name, mother's name, date of birth, sex, and municipality of residence.
- Number of linked records over the defined cut-off point: 7,802,044 (number of people hospitalized among those registered at CadÚnico)

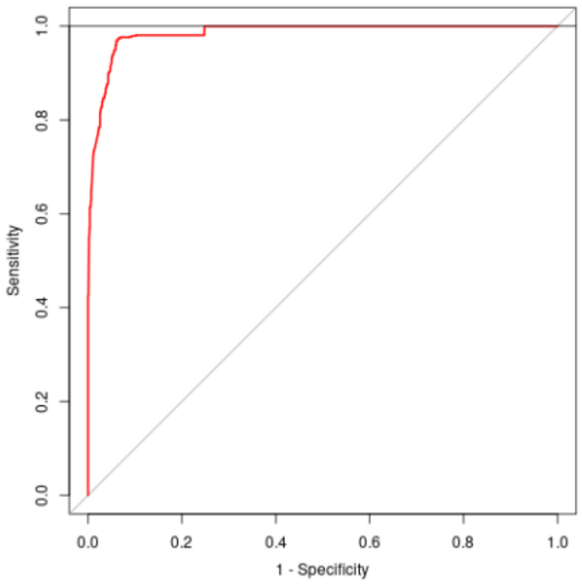

eFigure 1- ROC curve of the 100 million Brazilian Cohort and SIH (2001-2018) linkage
Source: Developed by the CIDACS Data Production Center.

© 2026 Bonfim CB et al. *JAMA Network Open.*

eMethods 2. Propensity score: definition, estimation, summary, and support graphs

PS is employed when comparing intervention and non-intervention groups, as the allocation of these groups is not randomized[12]. This lack of randomization can result in imbalanced distribution of covariates, leading to biased estimates[4]. By controlling for confounding factors, PS aids in correcting the estimation of the intervention's effect[5]. The propensity scores were obtained from logistic regression[5] to estimate the conditional probability of receiving BFP (eTable 1). Then, we assessed the common support graph (eFigure 2) and we compared the range of propensity scores among BFP and non-BFP groups. A summary of the propensity score stratified by BFP groups was shown in eTable 2a. Given the reduced area of common support, we conducted an additional analysis excluding individuals at the extremes of the propensity score distribution (<0.1 and >0.9), as recommended by Crump et al.[6] to address situations of limited overlap (eTable 3, eFigure 3).

eTable 1. Logistic regression to estimate propensity scores for receiving BFP according to covariables, N= 34,864,735

Variable	Odds Ratio (95% CI)	p value
**Sex**		
Male	Ref.	
Female	1.25 (1.24 - 1.26)	<.001
**Age group (years old)**		
<10	Ref.	
10-24	0.78 (0.77, 0.79)	<.001
25-59	0.49 (0.48,0.50)	<.001
>60	0.05 (0.04, 0.06)	<.001
**Education Level (years of education)**		
Never been study	Ref	
Preschool	1.21 (1.20-1.22)	<.001
Primary school or less (<= 5 years)	1.30 (1.29-1.31)	<.001
Junior high school (6- 10 years)	1.39 (1.38-1.40)	<.001
High school (10-12 years)	1.01 (1.00-1.02)	<.001
College/university (>=13 years)	0.31 (0.30-0.32)	<.001
**Race**		
White	Ref.	
Asian	1.13 (1.12-1.15)	<.001
Black	1.34 (1.33-1.35)	<.001
Indigenous	2.01 (1.98-2.04)	<.001
Parda	1.22 (1.21-1.23)	<.001
**Location of residence**		
Urban	Ref.	
Rural	0.93 (0.92-0.94)	<.001
**Brazilian regions**		
Southeast	Ref.	
Northeast	1.01 (1.00-1.02)	.003
Midwest	0.68 (0.67-0.69)	<.001
South	0.56 (0.55-0.57)	<.001
North	0.98 (0.97-0.99)	<.001
**Household characteristics**		
**Water supply**		
Public Network (running water)	Ref.	
Well- natural sources- or other	1.25 (1.34-1.26)	<0.001

© 2026 Bonfim CB et al. *JAMA Network Open.*

**Waste**		
Public collection system	Ref.	
Burned- buried- outdoor disposal- o other	1.10 (1.09-1.11)	<.001
**Sanitation**		
Public network	Ref.	
Septic tank	1.00 (0.99-1.02)	.004
Homemade septic tank	0.99 (0.98-1.00)	<.001
Ditch or other	1.38 (1.37-1.39)	<.001
**Construction materials**		
Bricks/ cement	Ref.	
Wood- other vegetal materials- and other	1.35 (1.34-1.36)	<.001
**Isolation**		
Live with someone else	Ref.	
Live alone	0.59 (0.58-0.60)	<.001
**Year of registration at CadÚnico**		
2008	Ref.	
2009	0.86 (0.85-0.87)	<.001
2010	0.62 (0.61- 0.63)	<.001
2011	0.33 (0.32-0.34)	<.001
2012	0.22 (0.21-0.23)	<.001
2013	0.17 (0.16-0.18)	<.001
2014	0.11 (0.10-0.12)	<.001
2015	0.07 (0.06-0.08)	<.001

eFigure. 2. Distribution of the propensity score in the sample, 2008-2015

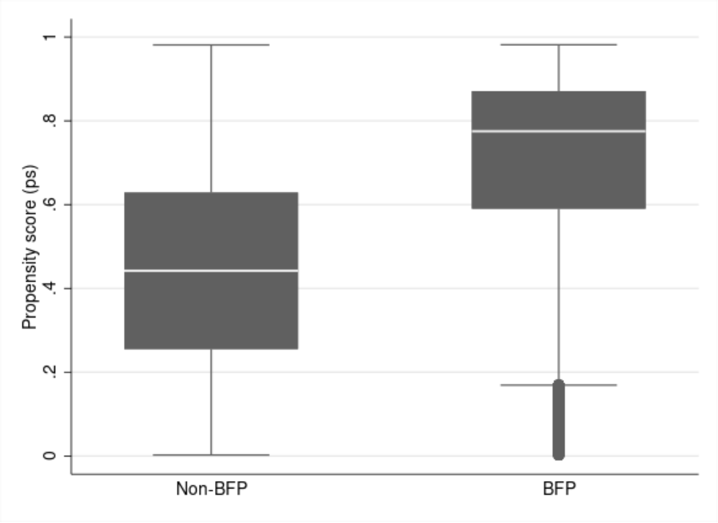

© 2026 Bonfim CB et al. *JAMA Network Open.*

eFigure 3. Cumulative Distribution Balancing Plot - Balance between variables before and after IPTW, 2008-2015

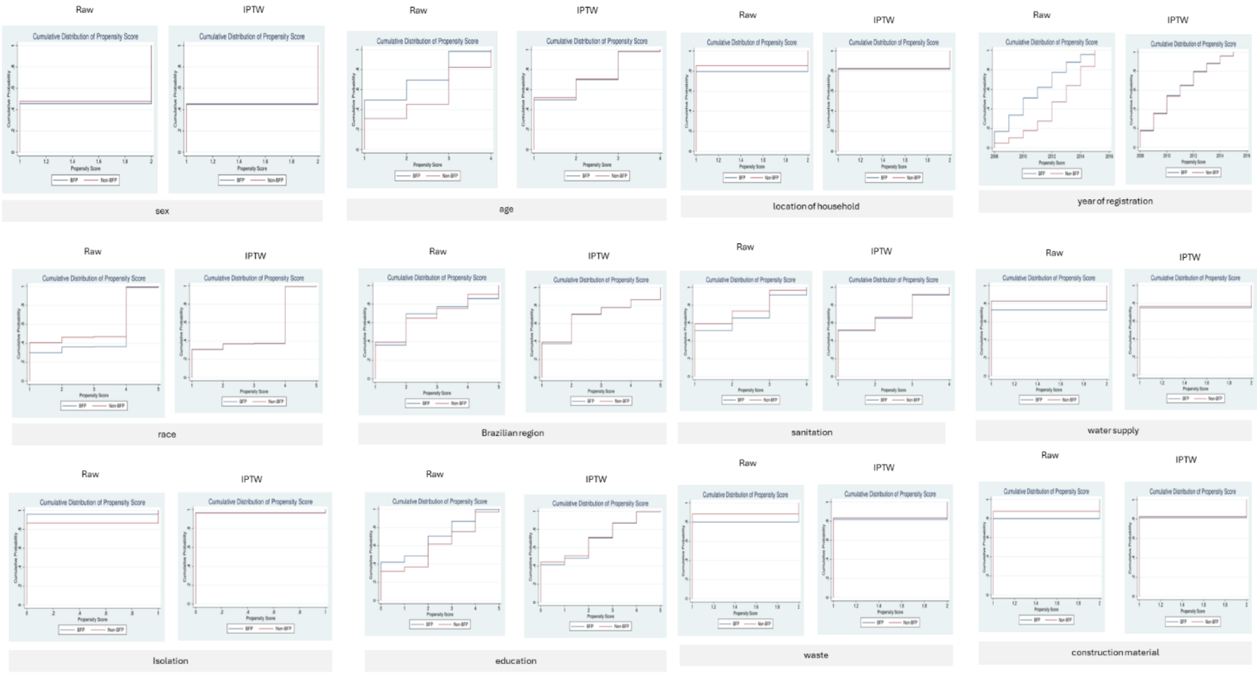

eTable 2. Propensity score description in accordance with the confounding covariates observed, Brazil, 2008 to 2015, N= 34,864,735

**Propensity score**	**BFP**<br>**N= 21,230,441**	**Non- BFP**<br>**N= 13,634,294**
Average	0.714	0.443
Standard deviation	0.195	0.246
Minimum	0.003	0.002
Maximum	0.982	0.981

eTable 3. Propensity score description in accordance with the confounding covariates observed after exclusion of individuals with extreme values (<0.1 - >0.9), Brazil, 2008 to 2015, N= 29,909,313

**Propensity score**	**BFP**<br>**N= 18,069,079**	**Non- BFP**<br>**N= 11,840,234**
Average	0.684	0.485
Standard deviation	0.182	0.208
Minimum	0.100	0.100
Maximum	0.900	0.900

© 2026 Bonfim CB et al. *JAMA Network Open.*

eMethods 3. Sensitivity analyses

First, we estimated Poisson regression models (eTable 4 in Supplement 1). Second, we applied a kernel matching approach as an alternative method (eTable 5 and eTable 6 in Supplement 1)[5]. Third, we estimated crude and doubly adjusted Cox Regression using covariates and IPTW (eTable 7 in Supplement 1). Fourth, we repeated the main model after excluding individuals with extreme PS values, considering potential impacts on groups balance[6] (eTable 8 in Supplement 1). Fifth, we repeated the main model after excluding individuals hospitalized within the first year of registration in the CadÚnico to minimize reverse causality (eTable 9 in Supplement 1). Additional analyses were conducted to assess effect modification (eTable 10 in Supplement). Models including and excluding the interaction term were compared using the Akaike Information Criterion (AIC) and the Bayesian Information Criterion (BIC)[7] (eTable 10 in Supplement 1). Based on established guidelines, an AIC difference of less than 2 was interpreted as indicating that the addition of the interaction term did not meaningfully improve model fit[7]. We also checked the proportional hazards assumptions for the main Cox model using proportional hazards test[8] (eTable11 in Supplement 1) and Kaplan-Meier curve[8] (eFigure 4 in Supplement 1).

eTable 4. Incidence rate ratio of Bolsa Família Program participation with psychiatric hospitalizations- 2008-2015. N= 34,864,735

**Confounder adjustment**	**IRR (95% CI)**	**p value**
Poisson adjusted with IPTW[a]		
Non-BFP	1.00	
BFP	0.78 (0.77 – 0.80)	<.001

Abbreviations: BFP - Bolsa Família Program; IRR - incidence rate ratio; CI - confidence interval; IPTW - inverse probability of treatment weighting.

[a] IRR estimated with IPTW given sex, age, race, education level, household characteristics (water supply, waste, sanitation, and construction materials), living alone, crowding, Brazilian region, location of residence, and year of CadÚnico registration.

eTable 5. ATT of psychiatric hospitalizations for BFP receipt between 2008 and 2015 using Kernel matching

	Kernel Weighting ATT[a] (95% CI)
ATT	-0.0004246 (-0.0004501 - -0.0003991)
N	38,572,143

[a] Average treatment effect on the treated (ATT) estimated using kernel matching (PS variables).

© 2026 Bonfim CB et al. *JAMA Network Open.*

eTable 6. Association of Bolsa Família Program participation with psychiatric hospitalizations adjusted for kernel matching- 2008-2015. N= 38,572,143

**Confounder adjustment**	**HR (95% CI)**	**p value**
Cox adjusted with kernel matching[a]		
Non-BFP	1.00	
BFP	0.42 (0.41- 0.43)	<.001

Abbreviations: BFP - Bolsa Família Program; HR - Hazard Ratio; CI - confidence interval.

[a] HR estimated with kernel matching given sex, age, race, education level, household characteristics (water supply, waste, sanitation, and construction materials), living alone, crowding, Brazilian region, location of residence, and year of CadÚnico registration.

eTable 7. Crude and adjusted association of Bolsa Família Program participation with psychiatric hospitalizations, 2008-2015.

**Confounder adjustment**	**N**	**HR (95% CI)**	**p value**
Cox with no adjustment			
Non-BFP	38,572,143	1.00	<.001
BFP		0.42 (0.41- 0.43)	
Cox with adjustement[a]			
Non-BFP	34,864,735	1.00	<.001
BFP		0.81 (0.79- 0.83)	

Abbreviations: BFP - Bolsa Família Program; HR - Hazard Ratio; CI - confidence interval.

[a] HR doubly adjusted by IPTW and sex, age, race, education level, household characteristics (water supply, waste, sanitation, and construction materials), living alone, crowding, Brazilian region, location of residence and year of CadÚnico registration.

© 2026 Bonfim CB et al. *JAMA Network Open.*

eTable 8. Association of Bolsa Família Program participation with psychiatric hospitalizations adjusted for IPTW after exclusion of individuals with Propensity Score extreme values (<0.1 - >0.9), 2008-2015. N= 29,909,313

Confounder adjustment	HR (95% CI)	p value
Cox adjusted with IPTW[a]		
Non-BFP	1.00	
BFP	0.78 (0.77- 0.80)	<.001

Abbreviations: BFP - Bolsa Família Program; HR - Hazard Ratio; CI - confidence interval; IPTW - inverse probability of treatment weighting.

[a] HR estimated with IPTW given sex, age, race, education level, household characteristics (water supply, waste, sanitation, and construction materials), living alone, crowding, Brazilian region, location of residence, and year of CadÚnico registration.

eTable 9. Association of Bolsa Família Program participation with psychiatric hospitalizations adjusted for IPTW after exclusion of individuals hospitalized within the first year of registration in the CadÚnico, 2008-2015. N= 34,707,794

Confounder adjustment	HR (95% CI)	p value
Cox adjusted with IPTW[a]		
Non-BFP	1.00	
BFP	0.77 (0.75- 0.79)	<.001

Abbreviations: BFP - Bolsa Família Program; HR - Hazard Ratio; CI - confidence interval; IPTW - inverse probability of treatment weighting.

[a] HR estimated with IPTW given sex, age, race, education level, household characteristics (water supply, waste, sanitation, and construction materials), living alone, crowding, Brazilian region, location of residence, and year of CadÚnico registration.

© 2026 Bonfim CB et al. *JAMA Network Open.*

eTable 10 – Information Criteria–Based Model Comparison Evaluating the Inclusion of an Interaction Term.

Models	Observations	Log-likelihood	Degrees of freedom	AIC	BIC
**Race**					
Model 1 (main effect)	34,864,735	-1041706	5	2076524	2076601
Model 2 (main effect + interaction term)	34,864,735	-1041706	9	2076486	2076624
**Brazilian Deprivation Index**					
Model 1 (main effect)	34,860,073	-1041598	3	2067098	2067145
Model 2 (main effect + interaction term)	34,860,073	-1041598	5	2067068	2067145
**Availability of CAPS in the municipality**					
Model 1 (main effect)	34,864,735	-1040914	2	2081831	2081862
Model 2 (main effect + interaction term)	34,864,735	-1040912	3	2081831	2081877

AIC= Akaike Information Criterion; BIC= Bayesian Information Criterion

eTable 11 - Proportional Hazards (PH) Assumption test

	chi2	Degrees of freedom	p value
Global test	9155.14	1	<.001

© 2026 Bonfim CB et al. *JAMA Network Open.*

eFigure 4 – Kaplan-Meier curve representing time to psychiatric hospitalization by BFP

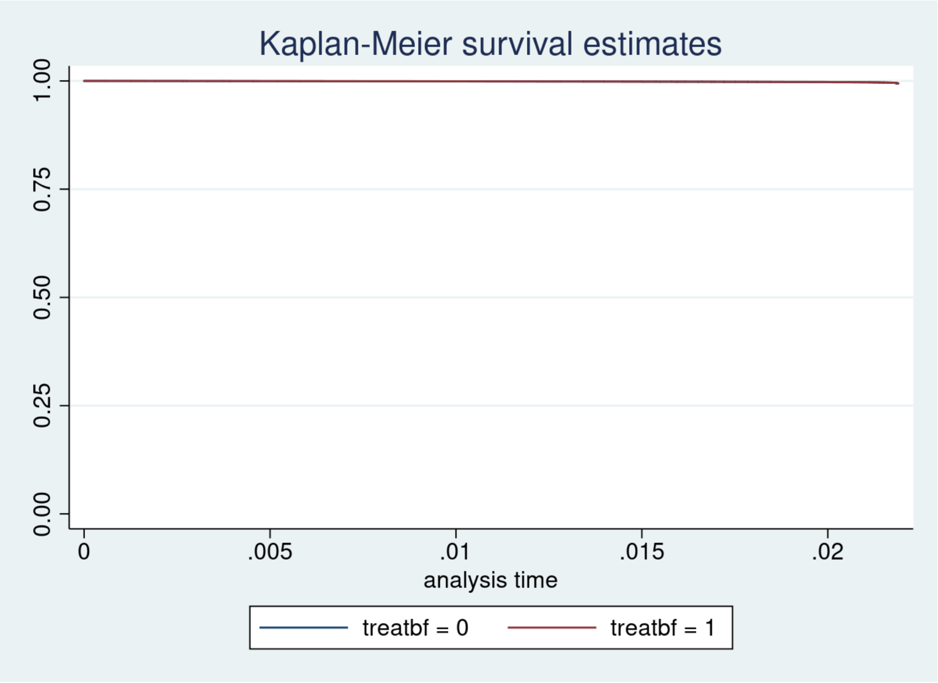

© 2026 Bonfim CB et al. *JAMA Network Open.*

eReferences

1 Almeida D- Gorender D- Ichihara MY- Sena S- Menezes L- Barbosa GCG- et al. Examining the quality of record linkage process using nationwide Brazilian administrative databases to build a large birth cohort. *BMC Med Inform Decis Mak.* 2020;20(1):173. doi: 10.1186/s12911-020-01192-0

2 Barbosa GCG- Ali MS- Araujo B- Reis S- Sena S- Ichihara MYT- et al. CIDACS-RL: a novel indexing search and scoring-based record linkage system for huge datasets with high accuracy and scalability. *BMC Med Inform Decis Mak.* 2020;20(289). doi: 10.1186/s12911-020-01285-w

3 Barreto ML- Ichihara MY- Almeida BA- Barreto ME- Cabral L- Fiaccone RL- et al. The Center for Data and Knowledge Integration for Health (CIDACS): Linking health and social data in Brazil. *Int J Popul Data Sci.* 2019; 4(2):1-12. doi: 10.23889/ijpds.v4i2.1140

4 Williamson E- Morley R- Lucas A- Carpenter J. Propensity scores: from naive enthusiasm to intuitive understanding. *Stat Methods Med Res.*2012; 21(3):273-293. doi: 10.1177/0962280210394483

5 Ali MS- Prieto-Alhambra D- Lopes LC- Ramos D- Bispo N- Ichihara MY- et al. Propensity Score Methods in Health Technology Assessment: Principles- Extended Applications- and Recent Advances. *Front Pharmacol.* 2019; 10(973): 1-19. doi: 10.3389/fphar.2019.00973

6 Crump RK, Hotz VJ, Imbens GW, Mitnik OA. Dealing with limited overlap in estimation of average treatment effects. *Biometrika.* 2009;96(1):187-199. doi: 10.1093/biomet/asn055

7 Burnham KP, Anderson DR. Model selection and multimodel inference: a practical information-theoretic approach. 2nd ed. New York: Springer; 2002.

8 Therneau TM. Modeling survival data: extending the Cox Model. New York: Springer; 2000

© 2026 Bonfim CB et al. *JAMA Network Open.*
