# Supplementary material for: Cash Transfers and Psychiatric Hospitalization for At-Risk Populations in Brazil
Source: JAMA Netw Open. 2026 Apr 13;9(4):e266571. doi: 10.1001/jamanetworkopen.2026.6571 (PMC13077514; doi:10.1001/jamanetworkopen.2026.6571)
Supplement: Supplement 2. — Data Sharing Statement [file jamanetwopen-e266571-s002.pdf]

Data Sharing Statement

Bonfim. Cash Transfers and Psychiatric Hospitalization for At-Risk Populations in Brazil. *JAMA Netw Open*. Published April 13, 2026. doi:10.1001/jamanetworkopen.2026.6571

Data
Data available: No

Additional Information
Explanation for why data not available: The data described in the manuscript, code book and analytical code will be made available upon request to the corresponding author, email: cidacs.curadoria@fiocruz.br. The data supporting the findings presented in this study were obtained from the Centre of Data and Knowledge Integration for Health (CIDACS). Full access to the data is restricted due to its sensitive nature and the exclusive licensing agreement for its use in this study. The privacy regulations set by the Brazilian Ethics Committee prohibit public availability of the data. However, upon reasonable request and provided all ethical and legal requirements are met, the institutional data curation team can make the data available. Further information can be obtained at https://cidacs.bahia.fiocruz.br/acesso-aos-dados/.
